# The low-density lipoprotein receptor-related protein 5 *(LRP5) 4037C>T* polymorphism: candidate for susceptibility to type 1 diabetes mellitus

**DOI:** 10.20945/2359-3997000000057

**Published:** 2018-08-01

**Authors:** Karla Simone Costa de Souza, Marcela Abbott Galvão Ururahy, Yonara Monique da Costa Oliveira, Melina Bezerra Loureiro, Heglayne Pereira Vital da Silva, Raul Hernandes Bortolin, André Ducati Luchessi, Ricardo Fernando Arrais, Rosário Dominguez Crespo Hirata, Maria das Graças Almeida, Mário Hiroyuki Hirata, Adriana Augusto de Rezende

**Affiliations:** 1 Universidade Federal do Rio Grande do Norte Universidade Federal do Rio Grande do Norte Departamento de Análises Clínicas e Toxicológicas Natal RN Brasil Departamento de Análises Clínicas e Toxicológicas, Universidade Federal do Rio Grande do Norte (UFRN), Natal, RN, Brasil; 2 Universidade Federal de Campina Grande Universidade Federal de Campina Grande Centro de Educação e Saúde Cuité PB Brasil Centro de Educação e Saúde, Universidade Federal de Campina Grande (UFCG), Cuité, PB, Brasil; 3 Universidade Federal do Rio Grande do Norte Universidade Federal do Rio Grande do Norte Departamento de Pediatria Natal RN Brasil Departamento de Pediatria, Universidade Federal do Rio Grande do Norte (UFRN), Natal, RN, Brasil; 4 Universidade de São Paulo Universidade de São Paulo Departamento de Análises Clínicas e Toxicológicas São Paulo SP Brasil Departamento de Análises Clínicas e Toxicológicas, Universidade de São Paulo (USP), São Paulo, SP, Brasil

**Keywords:** Type 1 diabetes mellitus, LRP5, 4037C>T, rs3736228

## Abstract

**Objective:**

The present study has investigated the association between low-density lipoprotein receptor-related protein 5 *(LRP5) 4037C>T* polymorphism and type 1 diabetes mellitus (T1DM) susceptibility in a Brazilian population.

**Subjects and methods:**

A total number of 134 T1DM patients and 180 normoglycemic individuals (NG) aged 6-20 years were studied. Glycated hemoglobin and glucose levels were determined. Genotyping of *LRP5 4037C>T* (rs3736228) was performed.

**Results:**

T1DM patients showed poor glycemic control. Genotypes in the codominant (CT: OR = 2.99 [CI 95%: 1.71-5.24], *p* < 0.001; TT: OR = 5.34 [CI 95%: 1.05-2702], *p* < 0.001), dominant (CT + TT: OR = 3.16 [CI 95%: 1.84-5.43], *p* < 0.001) and log-additive (OR = 2.78 [CI 95%: 1.70-4.52], *p* < 0.001) models, and *LRP5 4037T* allele (OR = 2.88, [CI 95%: 1.78-4.77], *p* < 0.001) were associated with an increased risk of developing T1DM. *LRP5 4037CT* and CT+TT carriers in T1DM group showed higher concentrations of serum glucose and glycated hemoglobin when compared with CC carriers.

**Conclusion:**

The *LRP5 4037C>T* may represent a candidate for T1DM susceptibility, as well as poor glycemic control.

## INTRODUCTION

Type 1 diabetes mellitus (T1DM) is a chronic autoimmune disorder that develops in susceptible individuals by environmental and genetic factors (1-3). Among the genetic factors, besides the HLA region on chromosome 6q21, which contributes to approximately 40% of T1DM development, more than 50 non-HLA genes significantly increase the risk of T1DM (1,2,4). Throughout nearly four decades since this discovery, researchers have investigated other genetic *loci,* in addition to HLA, that could contribute to the risk of T1DM (5,6).

Among several other mapped *loci* are those on chromosome 11q13.4 (7,8). Within the chromosome region linked to and associated with T1DM, the member of the LDL receptor family, low-density lipoprotein receptor-related protein 5 *(LRP5)* has been identified (5,7,8). *LRP5* is composed of a rapidly expanding number of structurally related proteins which serve a variety of functions in lipid metabolism and signal transduction to participate in cell proliferation (7-9). In addition, LRP5 is a membrane protein found in many tissues and cells, such as pancreatic beta cells, being essential for the insulin hormone production signaling pathway (10).

Fujino and cols. (10) studied the role of *LRP5* in pancreatic beta cells through animal model and found that the lack of the gene encoding LRP5 disrupts β-catenin activity by deleting the *LRP5*, a co-receptor involved in β-catenin-dependent wingless-ints (Wnt) signaling, greatly impairing beta cell proliferation, as well as glucose-stimulated insulin secretion, which coincides with reduced levels of intracellular ATP and calcium in response to glucose. Moreover, polymorphisms and mutations in the *LRP5* are associated with osteoporosis, impaired glucose, increased risk for obesity, type 2 diabetes mellitus, and other metabolic diseases (7-9,11,12).

Studies performed in diabetic families for scan of candidate genes of the T1DM, have indicated that *LRP5* missense mutations can trigger a non-coding LRP5, which could be associated with dysfunction in activity beta cells, lack of insulin production, and consequently T1DM development (7-9). However, to the best our knowledge, there are only a few studies associating the *LRP5* polymorphism and T1DM susceptibility (9).

Therefore, considering missense polymorphism *LRP5 4037C>T,* characterized by a substitution of alaninevaline amino acids in position 1330 (Ala1330Val), this study aimed to investigate the association between *LRP5 4037C>T* and T1DM susceptibility in children and adolescents in a Brazilian population.

## SUBJECTS AND METHODS

### Study population

We recruited 134 T1DM patients between 6 and 20 years old from the Hospital Onofre Lopes (HUOL) of the Federal University of Rio Grande do Norte (UFRN), Natal, RN, Brazil from January 2010 to June 2011. T1DM diagnoses were in accordance to the American Diabetes Association (ADA) criteria (3). All patients had a typical history of hyperglycemia and required insulin treatment continuously upon diagnosis. The study was conducted in accordance with the guidelines set by the Ethics in Research Committee of HUOL/UFRN, which complies with the Declaration of Helsinki (protocol number 704 310). One hundred eighty unrelated subjects with no previous diagnosis of T1DM, with fasting serum glucose ≤ 99 mg/dL (Normoglycemic Group - NG) and the same age range were recruited in local public schools of Natal, RN, Brazil. Informed consent was obtained from all T1DM and NG subjects or their parents. After taking a medical history and performing a physical examination, blood samples were collected for biochemical analysis and DNA extraction.

### Laboratory procedures

Glycemic control was assessed by the determination of glycated hemoglobin in total blood and serum glucose concentration. Both tests were performed using LABTEST kits (Lagoa Santa, Brazil) and LABMAX PLENNO equipment (LABTEST) for glucose and RA 50 spectrophotometer (Bayer Diagnostics, Dublin, Ireland) for glycated hemoglobin.

### *LRP5* genotyping

DNA was obtained from peripheral blood mononuclear cell (PBMC), previously isolated by density gradient centrifugation, using Ficoll-Hypaque (Sigma-Aldrich, MO, USA; density 1.077 g/mL). DNA was extracted using the commercial kit Illustra Triple Prep® (GE Healthcare, Little Chalfont, Buckinghamshire, UK), according to the manufacturer's protocol.

DNA integrity was evaluated by electrophoretic separation on a 0.8% agarose gel in TBE buffer (pH = 8.0) and stained with GelRed™ (Uniscience, São Paulo, SP, Brazil). DNA was quantified (A260nm) and had its purity (A260/A280) assessed by ultraviolet spectrophotometry, through the ND-1000 spectrophotometer (NanoDrop Technologies Inc., Wilmington, DE, USA).

*LRP5 4037*C>T (rs3736228) polymorphism was performed by TaqMan® allelic discrimination on a 7500 Fast Real-Time PCR System (Applied Biosystems, Foster City, CA, USA). *LRP5 4037*C>T was detected using the Applied Biosystems TaqMan® pre-designed assay C_25752205_10.

### Statistical analysis

Distribution of variables was analyzed by the Kolmogorov-Smirnov test. Variables with normal distributions were subjected to a t-test, and those skew-distributed were analyzed by Mann-Whitney's test. Differences between the sex, Hardy-Weinberg equilibrium, and allele/genotype frequencies were tested by a χ^2^ analysis test. Those tests were performed with SPSS version 15.0 (SPSS Inc., Chicago, IL, USA).

The logistic regression model was applied to analyze the genotypes according to the genetic models: codominant, dominant, overdominant, recessive, and log-additive (13). These analyses were assessed according to the goodness of fit evaluated by Akaike information criterion (AIC) value and Bayesian information criterion (BIC) (14,15). Odds ratio (OR), confidence interval (CI) of 95%, and their corresponding *p*-values were calculated to evaluate the study variables and their influence on T1DM onset. For *LRP5 4037C>T* polymorphism, *p*-value was computed using the likelihood ratio test. For this analysis, the R program library SNPassoc version 2.12.2 (R Foundation for Statistical Computing, Vienna, Austria) (16) was used and the reference genotype was homozygous for the wild allele among controls.

Tests with *p*-values < 0.05 were considered statistically significant.

## RESULTS

Table 1 shows characteristics of NG and T1DM patients. As expected, serum glucose and glycated hemoglobin values were significantly higher in the T1DM group compared to NG individuals (*p* < 0.001). Furthermore, glucose and glycated hemoglobin values in the T1DM group were higher than those recommended by the ADA for good glycemic control in the same age range of the studied individuals (glycated hemoglobin: ≥ 7.5%).

**Table 1:** Characteristics of normoglycemic and diabetic patients

Variables	NG n = 180	T1DM n = 134	*p*-value
Biodemographic characteristics			
Sex, Female, %	57.8	58.2	0.939
Age, years	11.0 (9.0-15.0)	12.0 (9.0-15.0)	0.876
Age at diagnosis, years	−	6.5 ± 3.5	−
Time since diagnosis, years	−	4.0 (2.0-7.0)	−
Glycemic control			
Glucose, mg/dL	79.0 (73.3-85.8)	208.0 (130.0-316.0)	**< 0.001**
Glycated hemoglobin, %	5.6 (4.8-6.4)	9.6 (7.9-12.3)	**< 0.001**

Results are expressed as mean ± standard deviation or median (interquartile range). n: number of individuals; NG: normoglycemic group; T1DM: type 1 diabetes mellitus group. Significant *p*-values are shown in bold.

The Hardy-Weinberg equilibrium was verified. Ten percent of the samples were re-genotyped at random to assure scoring quality, and all results were consistent.

Allele frequency for *LRP5 4037C>T* is demonstrated in Table 2. A significant increase in the frequency of the *LRP5 4037*T allele was found in T1DM compared to the NG group (*p* < 0.001).

**Table 2:** Allele frequencies and genotype distribution of *LRP5 4037C>T* polymorphism in the studied groups according to the genetic model

Polymorphism		NG n (%)	T1DM n (%)	OR (95% CI)	*p*-value	AIC
*LRP5 4037C>T*	Allele					
	C	331 (91.9)	214 (79.9)	1.00		
	T	29 (8.1)	54 (20.1)	2.88 (1.78-4.77)	**< 0.001**	−
	Genetic Model					
	Codominant					
	CC	153 (85.0)	86 (64.2)	1.00		
	CT TT	25 (13.9) 2 (1.1)	42 (31.3) 6 (4.5)	2.99 (1.71-5.24) 5.34 (1.05-27.02)	**< 0.001**	415.8
	Dominant					
	CC	153 (85.0)	86 (64.2)	1.00	**< 0.001**	414.3
	CT+TT	27 (15.0)	48 (35.8)	3.16 (1.84-5.43		
	Recessive					
	CC+CT	178 (98.9)	128 (95.5)	1.00		
	TT	2 (1.1)	6 (4.5)	4.17 (0.83-21.00)	0.060	429.0
	Log-additive					
		180 (57.3)	134 (42.7)	2.78 (1.70-4.52)	**< 0.001**	414.1

NG: normoglycemic group; T1DM: type 1 diabetes mellitus group; n: number of individuals; OR: odds ratio; CI: confidence interval; AIC: Akaike's Information Criterion. Significant *p*-values are shown in bold.

Considering the genotype distribution of *LRP5* 4037C>T in the NG and T1DM groups according to the genetic model, a significant association with T1DM susceptibility was found for codominant *(p* < 0.001), dominant *(p <* 0.001) and log-additive *(p <* 0.001) models (Table 2).

In this study, also was investigate the influence of *LRP5 4037C>T* in the parameters of glycemic control. Significantly increased values of serum glucose and glycated hemoglobin were observed in the *LRP5 4037CT* [glucose mean: 230.2 mg/dL (*p* < 0.001); glycated hemoglobin mean: 10.3% (*p* = 0.017)] and CT+ TT carriers [glucose mean: 227.8 mg/dL (*p* < 0.001); glycated hemoglobin mean: 10.5%, (*p* = 0.002)] when compared to the CC genotype carriers [glucose mean: 98.9 mg/dL; glycated hemoglobin mean: 7.3%].

## DISCUSSION

T1DM is characterized by autoimmune destruction of pancreatic beta cells with multiple genes involved, and among them is *LRP5* (3,7-9). The relationship of this gene with T1DM was found in 1998 by Nakagawa and cols., that described only 10 polymorphisms of *LRP5* associated with susceptibility to T1DM, after to perform a genomic scanning in 707 diabetic families from the UK, USA, Italy, and Norway. However, data in the literature on this association remains scarce and for our knowledge the present study is the first to find the association between *LRP5 4037C>T,* T1DM susceptibility, and poor glycemic control.

On the other hand, the *LRP5 4037C>T* is recognized as one of the key polymorphism in associated with increase the risk of bone fracture and osteoporosis, as well as in the cardiovascular diseases (12,17-20). In addition, it has been reported that the *LRP5 4037C>T* is associated with susceptibility to type 2 diabetes mellitus and osteoporosis in postmenopausal women. In this diseases, the genotype CT/TT increases the risk of the developing them (12,21).

LRP5 is a member of the LDL receptor superfamily, which was originally cloned on the basis of its genetic association with T1DM in humans, and is found in the 5' region of the chromosome 11q13 (9). The binding of Wnt with LRP5-frizzled receptor triggers LRP5 signaling. This link leads to inactivation of the “complex of destruction” of β-catenin, formed by proteins of adenomatous polyposis coli (APC), axis inhibition protein (Axin), and glycogen synthase kinase 3 (GSK-3β). Since GSK-3β is degraded by the dishevelled protein, it inhibits the proteasomal degradation of β-catenin, which accumulates in the cytoplasm and then translocates to the nucleus where it associates with transcription factors such as transcription factor 7, which will control the transcription of target genes for beta cell proliferation (14,22,23). Though there were no mechanistic explanations as to how Wnt signaling was involved in the increased proliferation of beta cell mass and insulin production, studies suggest that *LRP5* is essential for these cells (10,14,22).

Several studies have speculated that hyperglycemia may be conferred by changes in *LRP5* expression levels or tissue expression specificity, since LRP5 signaling contributes to the glucose-induced insulin secretion in the islets of Langerhans, however this mechanism is unclear (10,22). These studies raised the possibility that polymorphisms would favor the negative regulation of transcription or specificity of this gene on pancreatic beta-cells, hampering glucose-induced insulin secretion (10,22). A study using an animal model indicated that mice deficient of *LRP5* had low levels of insulin-signaling proteins for mRNA expression, such as the insulin receptor. Impaired glucose tolerance, as well as high glucose concentrations were found, demonstrating the importance of *LRP5* in this signaling (10). In addition, studies have associated *LRP5* missense mutations with the T1DM onset, once these mutations could change the pancreatic beta cell signaling, impairing insulin secretion (7-9,22).

Our results show an association between *LRP5* 4037C>T polymorphism and T1DM susceptibility. Furthermore, individuals who were carriers of CT and CT + TT genotypes had higher concentrations of serum glucose and glycated hemoglobin than individuals with the wild-type homozygous genotype. Thus, according Iniesta and cols. (13), the significant association found in codominant, dominant, and log-additive models demonstrate that each additional copy of the variant allele increases the T1DM susceptibility. Additionally, only one copy of the mutated T allele is sufficient to contribute to poor glycemic control.

In conclusion, our results demonstrated an association of the T allele for the *LRP5 4037C>T* polymorphism with T1DM susceptibility in a Brazilian population, moreover the possible relation in the progressive failure of glycemic control.
